# Solution‐Processable Van Der Waals Heterojunctions on Silicon for Self‐Powered Photodetectors with High Responsivity and Detectivity

**DOI:** 10.1002/advs.202500027

**Published:** 2025-03-28

**Authors:** Yuansheng Ge, Da Lei, Chaojun Zhang, Quan Zhang, Jinlong Mu, Jing Li

**Affiliations:** ^1^ Hubei Key Laboratory of Pollutant Analysis & Reuse Technology College of Chemistry and Chemical Engineering Hubei Normal University Huangshi 435002 China; ^2^ College of Chemistry and Chemical Engineering Xinjiang Agricultural University East Road No. 311 Urumqi Xinjiang 830052 China; ^3^ Key Laboratory of Green and High‐end Utilization of Salt Lake Resources Qinghai Institute of Salt Lakes Chinese Academy of Sciences Xining 810000 China; ^4^ Key Laboratory of Bio‐inspired Smart Interfacial Science and Technology of Ministry of Education School of Chemistry Beihang University Beijing 100191 China; ^5^ NAURA Technology Group Co., Ltd. & Department of Mechanical Engineering Tsinghua University Tsinghua Park, Haidian District Beijing 100084 China

**Keywords:** 2D materials, metal‐organic framework, self‐powered photodetector, solution process

## Abstract

The high density of surface states on silicon has long impeded the development of high‐performance photodetectors, leading to excessive dark leakage currents that adversely affect responsivity and detectivity. Herein, an all‐solution‐processable method is presented for fabricating photodetectors through consecutive spray‐coating of a conductive metal‐organic framework (MOF, Cu_3_(HHTP)_2_) and metallic Ti_3_C_2_ MXene to form van der Waals dual junctions on a silicon substrate. The heterojunction configuration facilitates unidirectional electron‐hole separation within the Cu_3_(HHTP)_2_/Si interface with type I band alignment, while leveraging the potential barrier difference between the Cu_3_(HHTP)_2_/Si and Ti_3_C_2_/Cu_3_(HHTP)_2_ Schottky junctions. The Ti_3_C_2_/Cu_3_(HHTP)_2_/Si photodetector demonstrates outstanding photoelectric performance, operating in a self‐powered mode with a high specific detectivity of 1.63 × 10^12^ Jones and a large responsivity of 1.8 A W^−1^ under 365 nm illumination. It also exhibits an impressive on/off ratio exceeding 3.9 × 10^4^ at an incident light power density of 330 µW cm^−2^. Additionally, the photodetector maintains excellent responsivity across a broad wavelength range from 365 to 700 nm, spanning ultraviolet to visible light, and sets a new performance benchmark for MOF‐based photodetectors. This work introduces a straightforward, controllable approach for constructing high‐quality van der Waals junctions on semiconductor surfaces, enabling the fabrication of optoelectronic devices with enhanced performance.

## Graphical abstract

This work reports an all‐solution processable fabrication approach for fabricating Ti_3_C_2_T_x_/Cu_3_(HHTP)_2_/*n*‐Si heterojunction as self‐powered photodetectors through the consecutive spray coating a Cu_3_(HHTP)_2_ and Ti_3_C_2_T_x_ thin films on *n*‐type silicon surface, as formed self‐supplied photodetector reveals exceptional responsivity and detectivity. By addressing interfacial defects through band design and structural optimization, this work paves the way for new applications and innovations in optoelectronics.

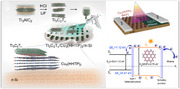

## 1 Introduction

Photodetectors are essential in optoelectronics, capable of converting incident radiation into electrical signals for further processing.^[^ ^1^, ^2^, ^3^, ^4^ ^]^ These devices show wide‐ranging applications across diverse fields, including optical communication, imaging, biological detection, chemical analysis, and healthcare monitoring.^[^ ^5^, ^6^, ^7^, ^8^, ^9^ ^]^ Self‐powered photodetectors, which operate without external power sources, represent a growing frontier in photodetection technology.^[^ ^10^ ^]^ Functioning in photovoltaic mode, photodetectors simultaneously generate power from the detected light. These detectors show high responsivity and detectivity, along with fast response times, rendering them ideal for rapid detection while with reduced device size, production costs, and energy consumption.^[^ ^11^, ^12^ ^]^

The physical mechanism underlying self‐powered photodetection involves (I) the generation of electron‐hole pairs via optical absorption of incident photons; (II) the separation of electron‐hole pairs by the built‐in electric field; (III) the collection of the photogenerated carriers through the external circuit.^[^ ^13^ ^]^ The performance of self‐powered photodetectors is largely determined by the quality of bulk heterolayers and their junctional interface.^[^ ^10^, ^14^ ^]^ For instance, several crystalline nanomaterials including graphene transition metal chalcogenides have been employed to optimize the height of the Schottky barrier for enhanced photodetector performance.^[^ ^15^ ^]^ However, these junction photodetectors still face limitations due to inefficient separation and transport of photo‐induced electron‐hole pairs. There remains a strong demand for junction materials with higher chemical tunability to precisely engineer photodetection performance.^[^ ^5^, ^16^ ^]^ Additionally, conventional semiconductor‐based photodetectors generally require complex microfabrication processes, such as high‐temperature diffusion and ion implantation, which reduce minority carrier lifetimes, degrade photoelectric performances, and increase production costs.^[^ ^17^, ^18^ ^]^

Two‐dimensional materials have been proven to be one of the most promising candidates for advanced photodetector construction, due to their strong light‐matter interactions, thickness‐dependent bandgaps, and high carrier mobility.^[^ ^5^ ^]^ 2D materials, such as transition metal dichalcogenides and InSe, have enormous potential in self‐powered ultraviolet to visible photodetectors.^[^ ^19^, ^20^ ^]^ Notably, the dangling bond‐free surfaces of 2D materials offer significant advantages in overcoming the strict lattice‐matching requirements, enabling the integration of high‐quality heterojunctions on a wide range of conventional bulk semiconductors, such as Si, Ge, GaAs, GaN, etc.^[^ ^14^, ^21^ ^]^ Among the emerging 2D materials, MXenes and metal‐organic frameworks (MOFs) stand out due to their versatile chemical composition and widely tunable electronic properties, providing a unique platform to finely tailor photodetector performance.^[^ ^22^, ^23^ ^]^ For instance, the surface functional groups on MXenes allow for the artificial engineering of work functions (ranging from 1.6 to 8.0 eV) and electronic band structures, offering additional pathways for optoelectronics,^[^ ^24^ ^]^ making them excellent Schottky electrode materials beyond conventional metals.^[^ ^25^, ^26^ ^]^ Compared to traditional inorganic materials, the diverse organic groups and metal nodes in 2D MOFs impart unique structural and electronic properties, offering systematic tunability in both structural flexibility and functional diversity.^[^ ^27^, ^28^, ^29^, ^30^ ^]^ Through structural modulation, triphenylene‐based electronically conductive MOFs can vary their band gap from 0.58 to 0.85 eV and increase their conductivity from 10^−6^ to 40 S cm^−1^.^[^ ^31^, ^32^ ^]^ Additionally, the natural porous morphology of MOFs is theoretically predicted to enhance strong photon capture, which enables the MOF‐based device to detect relatively low‐intensity photons, greatly significantly boosting light responsivity.^[^ ^33^ ^]^

In this work, we proposed an all‐solution processable method for constructing Ti_3_C_2_/Cu_3_(HHTP)_2_/*n*‐Si heterojunction as a self‐powered photodetector with high performance. The type I Cu_3_(HHTP)_2_/*n*‐Si heterojunction was first fabricated via liquid‐phase epitaxial growth of 2D conductive Cu_3_(HHTP)_2_ (HHTP = 2,3,6,7,10,11‐Hexahydroxybenzobenzene) MOF thin films on *n*‐Si surfaces. Ti_3_C_2_T_x_ MXenes nanosheets were then sprayed onto MOF/*n*‐Si thin films to form the Ti_3_C_2_/Cu_3_(HHTP)_2_/*n*‐Si photodetector. The as‐prepared photodetector exhibited outstanding photodetection performance, including an ultrahigh responsivity of 1.8 A W^−1^, a specific detectivity of 1.63 × 10^12^ Jones at zero external bias, and a prominent light on/off ratio exceeding 3.9 × 10^4^ under ultraviolet to visible light (365–700 nm).

## 2 Results and Discussion

**Figure 1:**
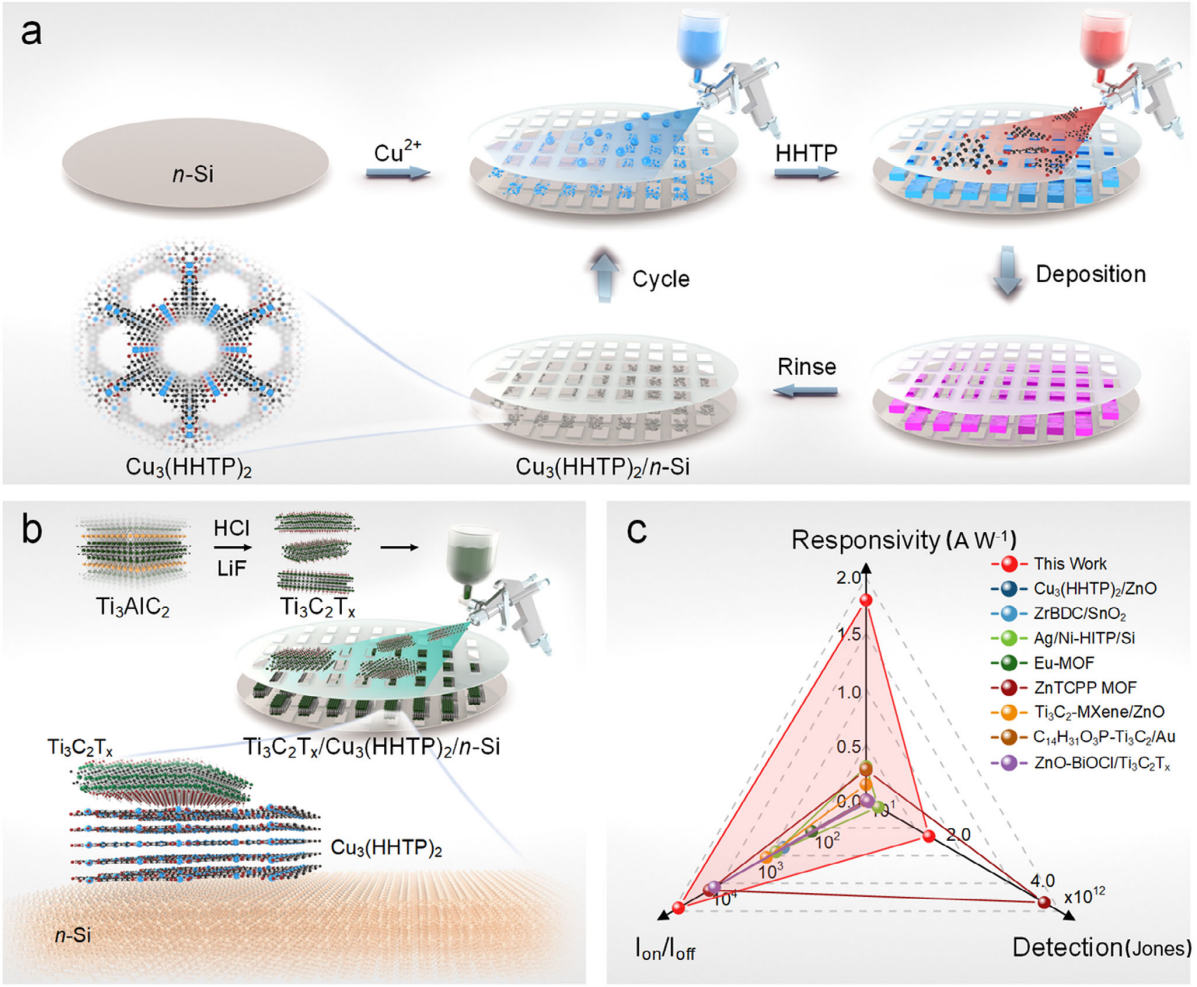
a) Synthesis schematic of Cu_3_(HHTP)_2_ on *n*‐Si. b) Schematic illustration of the preparation of Ti_3_C_2_/Cu_3_(HHTP)_2_/*n*‐Si photodetector. c) Comparison of performance parameters for various electrodes and attempts at using MXene or MOF materials in self‐powered photodetectors.^[^ ^34^, ^35^, ^36^, ^37^, ^38^, ^39^, ^40^, ^41^ ^]^

**Figure 2:**
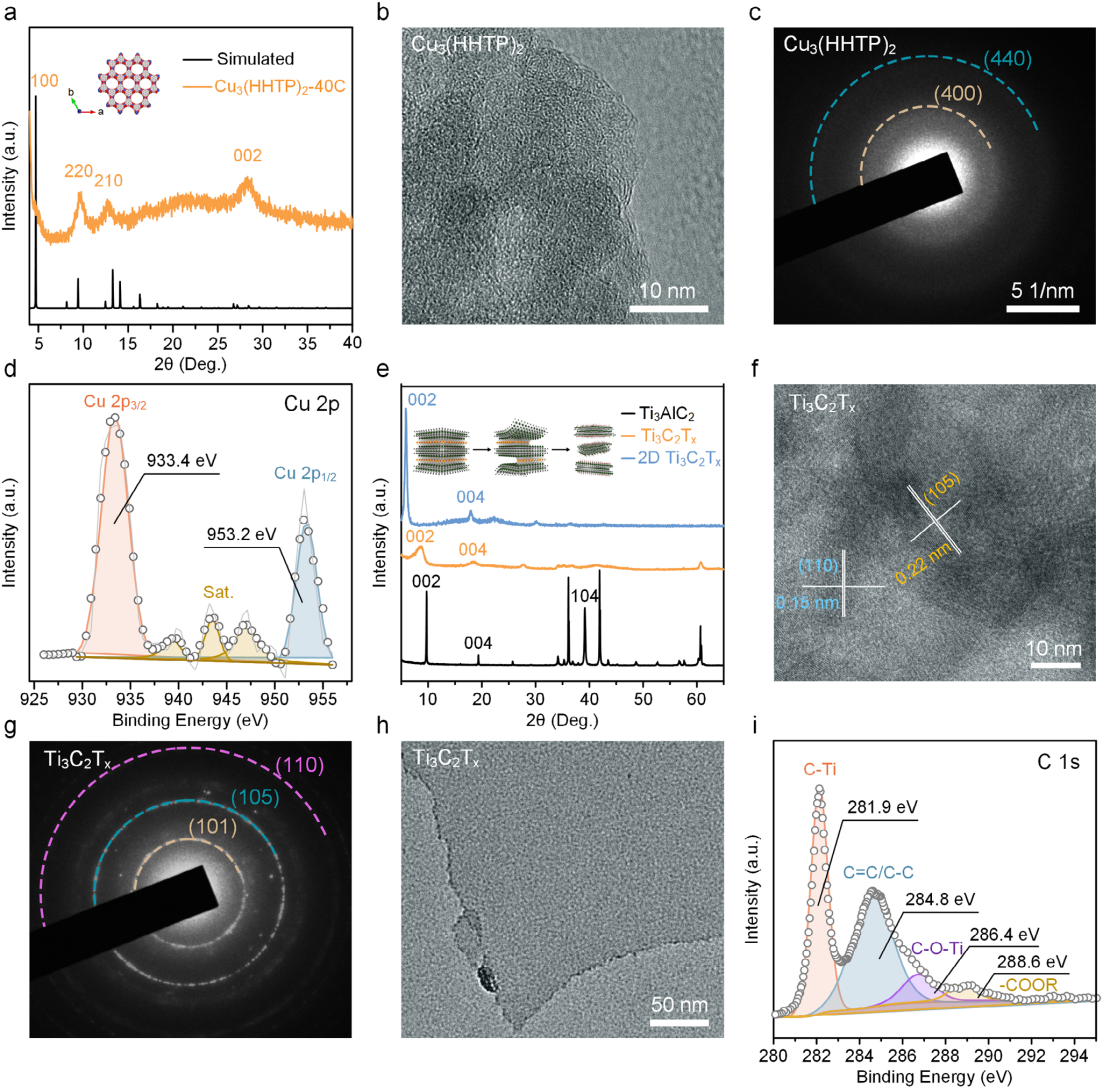
a) The simulated (black) and experimental (gold) XRD and simulated structural models of Cu_3_(HHTP)_2_‐40C thin film; b) HR‐TEM and c) SAED images of Cu_3_(HHTP)_2_‐40C thin film; d) Cu 2p XPS spectrum of Cu_3_(HHTP)_2_ thin film; e) XRD patterns of Ti_3_AlC_2_, multi‐layer Ti_3_C_2_, and single‐layer Ti_3_C_2_T_x_. The inset is a schematic illustration of interlayer structure before and after etching; f) HR‐TEM, g) SAED, and h) TEM images of Ti_3_C_2_T_x_; i) C 1s XPS spectrum of Ti_3_C_2_T_x_ nanosheets.

The fabrication of Ti_3_C_2_/Cu_3_(HHTP)_2_/*n*‐Si photodetector is summarized schematically in **Figure** **1**a,b, and the resulting Ti_3_C_2_/Cu_3_(HHTP)_2_/*n*‐Si dual‐junction structure photodetector demonstrates outstanding photoelectric performance (Figure 1c; Table S1, Supporting Information). Triphenylene‐based 2D MOFs were initially constructed on the *n*‐Si surface due to their strong metal‐ligand orbital hybridization and enhanced electrical conductivity. In contrast with the conventional powder‐formed MOF obtained via solvothermal synthesis, this work employed a layer‐by‐layer liquid phase epitaxy growth method to deposit thin MOF films on *n*‐Si surfaces, with the thickness of the MOF layer controlled by adjusting the number of coating cycles. The crystal structures of Cu_3_(HHTP)_2_ thin films were first analyzed using X‐ray diffraction (XRD). The unit cell parameters used for the simulation were: *a* = 21.75 Å, *b* = 221.75 Å, *c* = 6.6 Å, *α* = 90°, *β* = 90°, *γ* = 120°. The simulated structural model is shown in **Figure** **2**a, revealing that Cu_3_(HHTP)_2_ has a hexagonal structure with 2D slip parallel superposition. The XRD spectra (orange curve) of Cu_3_(HHTP)_2_‐40C film, grown layer‐by‐layer for 40 cycles, matched well with the XRD spectra (black curve) simulated for the AB stacking mode. The peaks observed at 2θ = 4.66 Å, 9.43 Å, 12.7 Å, and 28.25 Å, were ascribed to the (100), (220), (210), and (002) crystal planes, respectively. The XRD pattern indicated that the long‐range order of Cu_3_(HHTP)_2_‐40C film along the *C*‐axis was lower than that in the AB plane, a typical characteristic of layered 2D configuration.

As shown in Figure S1 (Supporting Information), the scanning electron microscope (SEM) and transmission electron microscopy (TEM) images of the Cu_3_(HHTP)_2_‐40C thin film displayed a dense and continuous morphology, with Cu_3_(HHTP)_2_ nanosheets uniformly distributed on the *n*‐Si substrate, forming a homogeneous film. Elemental analysis (Figure S2, Supporting Information) further confirmed that the elements C, O, and Cu were evenly distributed within the MOF film. Furthermore, high‐resolution transmission electron microscopy (HR‐TEM) was used to analyze the morphology and crystal structure of the Cu_3_(HHTP)_2_ film. As displayed in Figure 2b, the detached Cu_3_(HHTP)_2_ film showed well‐defined crystal lattices, indicating good crystallinity. In the selected area electron diffraction (SAED) image (Figure 2c), the diffraction rings revealed an oriented polycrystalline point array, while the (002) ring was absent. These results indicated the *C*‐axis oriented structural characteristics of the Cu_3_(HHTP)_2_ film.

To further investigate the chemical structure of Cu_3_(HHTP)_2_ thin films, XPS analysis was employed to determine the elemental composition and chemical valence states on the surface of the Cu_3_(HHTP)_2_ thin film. As displayed in Figure S3a (Supporting Information), the full XPS spectrum of the Cu_3_(HHTP)_2_‐40C thin film revealed characteristic peaks for C, O, and Cu. The high‐resolution O 1s XPS peak for Cu_3_(HHTP)_2_ appeared at 532.42 eV was attributed to O═C bonds (Figure S3b, Supporting Information). Additionally, the high‐resolution C 1s XPS peaks appeared between 284.8 and 288.8 eV, corresponding to C‐C or C═C, and C═O bonds in the Cu_3_(HHTP)_2_ structure (Figure S3c, Supporting Information), respectively. The high resolution of the Cu 2p XPS spectrum indicated the presence of both Cu (I) and Cu (II) cooperated with the HITP ligand, as evidenced by the two Cu 2p_3/2_ splitting peaks at 933.4 eV and 953.2 eV, along with satellite peaks, confirming the formation of the Cu_3_(HHTP)_2_ structure (Figure 2d).^[^ ^42^ ^]^

Ti_3_C_2_T_x_ nanosheets were obtained by exfoliating Ti_3_AlC_2_ using an ultrasound‐assisted method within HCl/LiF mixed solution. Crystal phase analysis was performed using XRD on Ti_3_AlC_2_ samples before and after etching (Figure 2e). The Ti_3_AlC_2_ samples before etching exhibited characteristic peaks at 9.58°, 19.25°, 34.09°, 36.73°, 39.0°, 41.74°, and 60.36°, corresponding to the (002), (004), (101), (103), (008), (104), and (110) crystal planes, respectively (PDF JCPDS#52‐0875). After the initial etching of Al atoms, the characteristic peaks of the (002) and (004) crystal planes in the accordion‐shaped Ti_3_C_2_ shifted to lower angles of 8.56° and 18.16°. In the original Ti_3_AlC_2_, Al, and Ti atoms were bonded by metallic bonds. As the etching time increased, the interaction between these atoms weakened or disappeared after the Al atoms were etched by HF acid. Functional groups such as ─OH, ─F, and ═O are anchored on Ti atoms, leading to an increased interlayer distance.

Following final ultrasonic etching, the characteristic peaks corresponding to the (002) and (004) crystal planes of the Ti_3_C_2_T_x_ nanosheets shifted further to 5.74° and 17.79°, illustrating that the van der Waals forces or hydrogen bonds between the Ti_3_C_2_T_x_ layers had been broken.^[^ ^43^ ^]^ The illustration in Figure 2e shows the structural variations of the Ti_3_AlC_2_ material before and after etching. As shown in Figure S4 (Supporting Information), the unetched Ti_3_AlC_2_ appeared as dense blocks (Figure S4a, Supporting Information). After HCl/LiF etching, the Al atoms were etched off, yielding an organ‐like layered structure (Figure S4b, Supporting Information). Repeated ultrasound‐assisted etching produced few‐layer or single‐layer Ti_3_C_2_T_x_ nanosheets with lateral sizes of 1–2 µm (Figure S4c, Supporting Information). Optical images showed that exfoliated Ti_3_C_2_T_x_ nanosheets revealed excellent dispersibility in solution, with no sedimentation observed even after weeks.

HR‐TEM images in Figure 2f and Figure S5 (Supporting Information) further displayed an interlayer distance of 0.96 nm, consistent with the observation in XRD. The well‐defined lattice spacings of 0.22 and 0.15 nm, along with the characteristic SAED pattern in Figure 2g, attest to the high crystallinity of the exfoliated Ti_3_C_2_T_x_ nanosheets.^[^ ^44^ ^]^ SEM and TEM images of the Ti_3_C_2_T_x_ nanosheets prepared by ultrasound‐assisted exfoliation (Figure 2h; Figure S5, Supporting Information) showed ultra‐thin morphological contrast with an average lateral size on the micrometer scale. The nanosheets exhibited characteristic 2D wrinkles and rolling, indicative of their high flexibility. Elemental mapping (Figure S6, Supporting Information) confirmed the uniform distribution of C, Ti, and O elements in the exfoliated nanosheets. The structure of the Ti_3_C_2_T_x_ nanosheets was analyzed using XPS (Figure S7a, Supporting Information), which detected C 1s, Ti 2p, Li 1s, O 1s, and F 2p signals. The high‐resolution C 1s spectrum (Figure 2i) revealed four characteristic peaks corresponding to Ti─C (281.9 eV), C─C (284.8 eV), C─O (286.4 eV), and C═O (288.6 eV) bonding configurations, indicating the presence of oxygen‐containing functional groups on the surface of the Ti_3_C_2_T_x_ nanosheets. The deconvoluted Ti 2p peak displayed a pair of peaks centered at 455.3 eV and 461.8 eV, corresponding to Ti 2p_1/2_ and Ti 2p_3/2_, respectively. The Ti 2p_1/2_ peak (Figure S7b, Supporting Information) consisted of Ti─C and Ti─O bonds at 456.5 eV and 458.5 eV, originating from the main framework structure and surface bonds of the Ti_3_C_2_T_x_ nanosheets.

**Figure 3:**
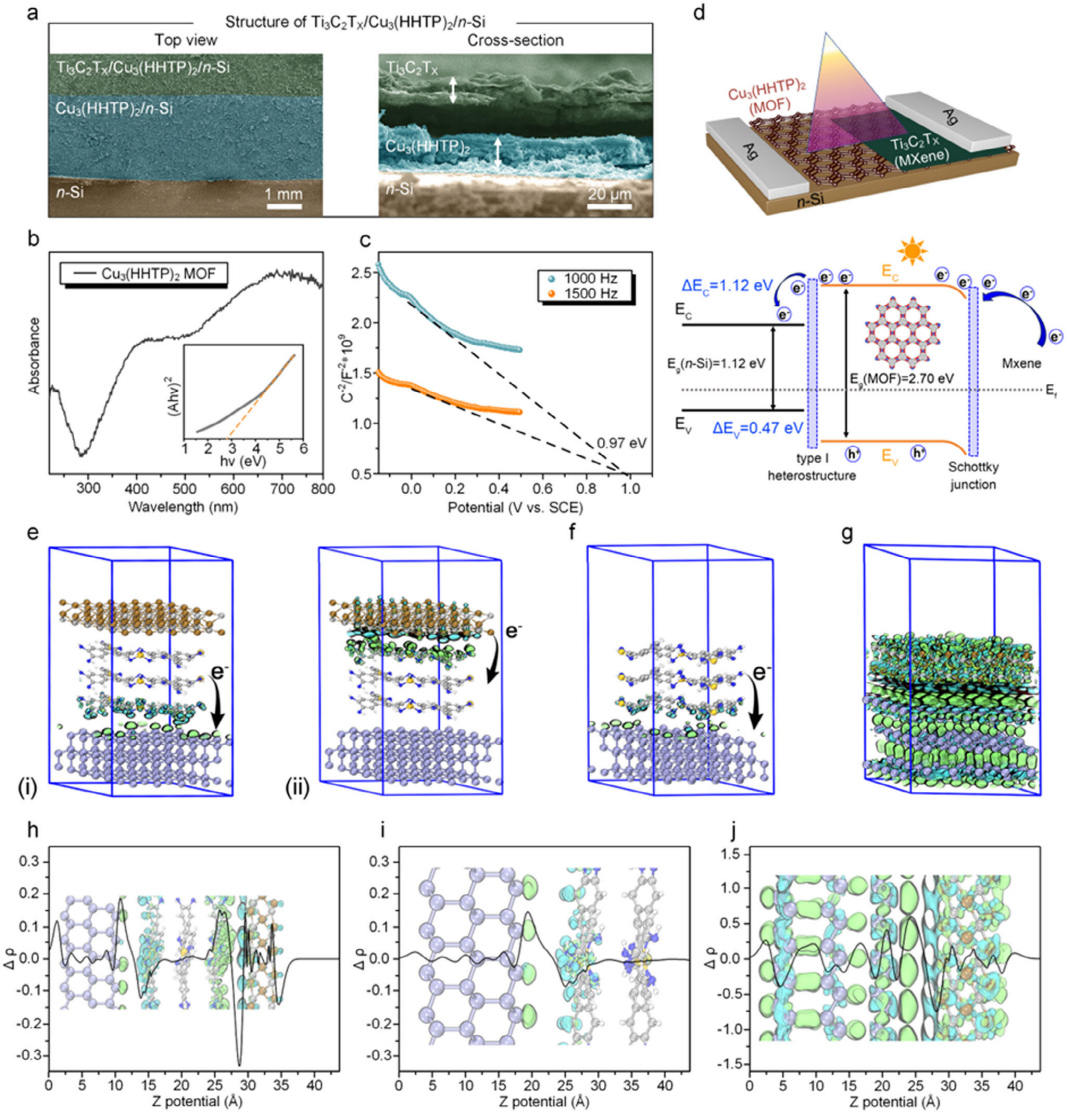
a) SEM images of Ti_3_C_2_T_x_/Cu_3_(HHTP)_2_/*n*‐Si with top view and cross‐section view; b) UV visible diffuse reflection of Cu_3_(HHTP)_2_ thin film (Kubelka Munk function curve in the lower right corner); c) The Mott–Schottky curve of Cu_3_(HHTP)_2_ thin film; d) Structure and band diagram of Ti_3_C_2_T_x_/Cu_3_(HHTP)_2_/*n*‐Si photodetector; e–g) First‐principle calculations of interlayer electron density differences between Ti_3_C_2_/Cu_3_(HHTP)_2_/*n*‐Si, Cu_3_(HHTP)_2_/*n*‐Si, and Ti_3_C_2_/*n*‐Si interfaces; h–j) Integral results of electron density difference in Ti_3_C_2_/Cu_3_(HHTP)_2_/*n*‐Si, Cu_3_(HHTP)_2_/*n*‐Si, and Ti_3_C_2_/*n*‐Si interfaces. The electron density distribution indicates the transfer of electrons from negative values (cyan isosurfaces) to positive values (green isosurfaces), respectively.

The homogeneity and thickness of the Ti_3_C_2_T_x_/Cu_3_(HHTP)_2_/*n*‐Si heterojunctions were investigated by top‐view and cross‐sectional focused SEM imaging.^[^ ^45^ ^]^ The fabrication process for the Ti_3_C_2_T_x_/Cu_3_(HHTP)_2_/*n*‐Si heterojunction involves the layer‐by‐layer liquid‐phase epitaxy of Cu_3_(HHTP)_2_ and consecutive spray coating of Ti_3_C_2_T_x_ on *n*‐Si substrate.^[^ ^46^ ^]^ The SEM images of the Ti_3_C_2_T_x_/Cu_3_(HHTP)_2_/*n*‐Si heterojunction revealed the sandwich structure of the device (**Figure** **3**a). A distinct interface between the Ti_3_C_2_T_x_, Cu_3_(HHTP)_2_, and Si substrate can be clearly observed. The SEM image in Figure 3a also shows that the Ti_3_C_2_T_x_ nanosheets were uniformly distributed on the surface of the MOF via the spraying approach. Figure S8 (Supporting Information) presents a cross‐sectional view of the Cu_3_(HHTP)_2_ layer grown on the Si surface 40 epitaxial growth cycles. The cross‐sectional SEM image of Ti_3_C_2_T_x_/Cu_3_(HHTP)_2_/*n‐*Si heterojunction in Figure 3a displays a clear layered structure, with the Ti_3_C_2_T_x_ layer sprayed on the MOF surface. This phenomenon can be attributed to the weak interfacial interactions caused by the layered structure and surface functional groups of Ti_3_C_2_T_x_ nanosheets. These morphological characterizations confirm that homogeneous and continuous heterojunctions have been successfully constructed via the solution‐processable spray‐coating approach.

For semiconductor materials, the edge energy level of the bandgap is a key indicator for assessing their photonic properties. UV–vis diffuse reflectance spectroscopy (UV–vis DRS) was performed with barium sulfate as the reference material in the solid state to analyze the absorption spectra and band gaps. As shown in Figure 3b, the Cu_3_(HHTP)_2_‐40C sample displayed strong absorption in the near‐UV and visible regions from 300 to 700 nm. The Tauc plot in Figure 3b with the inset in the lower‐right corner illustrating the corresponding Kubelka–Munk function curve. For the Cu_3_(HHTP)_2_‐40C sample, the (αhν)^2^ versus hν plot near the absorption edge (i.e., in the region where hν > Eg) exhibits a linear region, confirming a direct band gap nature, with a calculated value of 2.7 eV. To further study the intrinsic band structure of prepared Cu_3_(HHTP)_2_‐40C thin film, Mott–Schottky measurements were performed in darkness, and the flat band potential (E_fb_) was calculated for 0.97 eV versus SCE at 25 °C. The negative slope of the squared inverse capacitance‐potential (C^−2^–E) plots (Figure 3c) confirms that Cu_3_(HHTP)_2_ is a p‐type semiconductor, with the E_fb_ located near the valence band maximum (E_VB_). Therefore, the valence band maximum for the 2D Cu_3_(HHTP)_2_ was equivalent to 0.73 eV versus NHE. Based on the relationship E_CB_ = E_VB_–E_g_, the conduction band edge E_CB_ of the Cu_3_(HHTP)_2_ sample was calculated to be −1.97 eV.

The schematic diagram of the Cu_3_(HHTP)_2_/*n*‐Si heterojunction is displayed in Figure S9 (Supporting Information). Based on the band structure of Cu_3_(HHTP)_2_ (work function: 4.86 eV) and *n*‐Si (work function: 4.36 eV), they form a type I heterostructure. In such a configuration (Figure S9, Supporting Information), both the conduction band minimum (CBM) and valence band maximum (VBM) are positioned in the *n*‐Si, making it an ideal system for transferring photoexcited carriers. Upon illumination, the photoexcited electrons and holes in Cu_3_(HHTP)_2_ tend to flow into the *n*‐Si layer, thereby enhancing photo response performance by boosting the charge transfer dynamics.^[^ ^47^ ^]^ As shown in Figure 3d, further optimization of the band structure and work functions of the photodetector was employed to design the Ti_3_C_2_T_x_/Cu_3_(HHTP)_2_/*n*‐Si heterojunctions, enhancing the photo response performance. Cu_3_(HHTP)_2_ (work function: 4.86 eV) forms both type I heterojunction and Schottky contacts with *n*‐Si (work function: 4.36 eV) and Ti_3_C_2_T_x_ (work function: 4.56 eV), respectively.^[^ ^48^, ^49^, ^50^ ^]^ The underlying mechanism for the enhanced photo response performance of the Ti_3_C_2_T_x_/Cu_3_(HHTP)_2_/*n*‐Si is revealed through the energy band diagram. Ideally, a Schottky barrier is formed between Cu_3_(HHTP)_2_ and Ti_3_C_2_T_x_ with the formation condition of the work function of Ti_3_C_2_T_x_ less than the work function of Cu_3_(HHTP)_2_, which leads to the electric field in the barrier region to direct from the Ti_3_C_2_T_x_ layer to the Cu_3_(HHTP)_2_ end. This strong built‐in electric field, resulting from the large work function difference,^[^ ^51^ ^]^ drives electrons from Ti_3_C_2_T_x_ to Cu_3_(HHTP)_2_, depleting surface holes in Cu_3_(HHTP)_2_ and forming a negative space charge region. Meanwhile, in the type I heterostructure constructed by Cu_3_ (HHTP)_2_ and n‐Si, the photoexcited electrons tend to transfer to the semiconductor with a narrower bandgap, and thus the electrons transferred from Cu_3_(HHTP)_2_ to n‐Si. This transfer phenomenon in the heterostructure promotes the overall electron transfer in the photodetector, which enhances the intensity of photogenerated carriers enriched on n‐Si. Due to the smaller work function of *n*‐Si compared to Ti_3_C_2_T_x_, the electron injection barrier at the Cu_3_(HHTP)_2_/*n*‐Si interface is lower than that at the Cu_3_(HHTP)_2_/Ti_3_C_2_T_x_ interface.^[^ ^52^ ^]^ Consequently, electrons can pass through the Cu_3_(HHTP)_2_ layer from Ti_3_C_2_T_x_ layer to *n*‐Si, where they are collected by the electrode, generating a larger photocurrent due to increased electron collection at the *n*‐Si electrode.

To gain a more detailed understanding of the charge transfer processes between Ti_3_C_2_T_x_, Cu_3_(HHTP)_2_, and n‐Si, the first‐principles calculations were employed to investigate the interlayer electron density difference across the Ti_3_C_2_T_x_/Cu_3_(HHTP)_2_/*n*‐Si, Cu_3_(HHTP)_2_/*n*‐Si, and Ti_3_C_2_T_x_/*n*‐Si interfaces. In the Ti_3_C_2_T_x_/Cu_3_(HHTP)_2_/*n*‐Si trilayer model, the Cu_3_(HHTP)_2_ served as an intermediate layer facilitating electron transfer both from the upper Ti_3_C_2_T_x_ layer and toward the lower *n*‐Si layer (Figure 3e). The cyan and green isosurfaces in the electron density distribution (represented as the negative and positive values, respectively) at the Cu_3_(HHTP)_2_/*n*‐Si interface indicated a distinct electron transfer from Cu_3_(HHTP)_2_ to *n*‐Si, while a similar process occurs between Ti_3_C_2_T_x_ and Cu_3_(HHTP)_2_, indicating electron flow from Ti_3_C_2_T_x_ to Cu_3_(HHTP)_2_.

Further analysis of the Cu_3_(HHTP)_2_/*n*‐Si and Ti_3_C_2_T_x_/*n*‐Si systems revealed that the Cu_3_(HHTP)_2_/*n*‐Si interface exhibited a unidirectional charge transfer, whereas the Ti_3_C_2_T_x_/*n*‐Si interface displayed a more complex bidirectional electron transfer process (Figure 3f,g). To provide a more detailed explanation of Figure 3e–g, we analyzed the charge density difference maps and electron transfer mechanisms at different interfaces: Ti_3_C_2_/Cu_3_(HHTP)_2_/*n*‐Si, Cu_3_(HHTP)_2_/Si, and Ti_3_C_2_/Si. The charge density difference maps reveal charge redistribution at each interface, where cyan regions represent electron depletion and green regions indicate electron accumulation. In the Ti_3_C_2_/Cu_3_(HHTP)_2_/*n*‐Si structure (Figure 3e), significant charge transfer occurs from Ti_3_CT_2_T_x_ to Cu_3_(HHTP)_2_ and subsequently to Si, confirming a sequential electron transport pathway. This pathway facilitates effective charge separation and prevents recombination, leading to enhanced photodetection efficiency. In contrast, the Cu_3_(HHTP)_2_/Si interface (Figure 3f) shows localized charge accumulation at the interface, suggesting that although charge separation occurs, the efficiency of charge extraction is lower than that of the Ti_3_C_2_/Cu_3_(HHTP)_2_/*n*‐Si system. The Ti_3_C_2_/Si interface (Figure 3g) exhibits bidirectional charge transfer, indicative of Schottky barrier modulation, which aids in electron extraction but lacks the directional transport observed in the dual‐interface system. The integral charge density difference further confirms that the Ti_3_C_2_/Cu_3_(HHTP)_2_/*n*‐Si structure exhibits the most pronounced charge transfer, reinforcing the role of Cu_3_(HHTP)_2_ as an intermediate layer that enhances electron transport efficiency. Furthermore, integration of the electron density differences confirmed that electron transfer in the Ti_3_C_2_T_x_/Cu_3_(HHTP)_2_/*n*‐Si system was concentrated at the two interfaces, with a stronger electron transfer between Ti_3_C_2_T_x_ and Cu_3_(HHTP)_2_. In contrast, the Cu_3_(HHTP)_2_/*n*‐Si and Ti_3_C_2_T_x_/*n*‐Si systems show weaker unidirectional and stronger bidirectional charge transfer processes, respectively (Figure 3h–j). These results suggest that in the Ti_3_C_2_T_x_/Cu_3_(HHTP)_2_/*n*‐Si trilayer, once the electron‐hole pairs are generated in the Ti_3_C_2_T_x_ layer upon excitation, the electrons can easily transfer to the electrode via the Ti_3_C_2_T_x_‐to‐Cu_3_(HHTP)_2_‐to‐*n*‐Si pathway, enhancing the photovoltaic efficiency.

The specific roles of Cu_3_(HHTP)_2_ and Ti_3_C_2_T_x_ in the photoelectric detection process can be clearly understood from the density of states (DOS) analysis conducted. As shown in Figure S10 (Supporting Information), provides valuable insights into the electronic interactions and charge transport mechanisms within the heterojunction. Notably, in the energy range of −2 eV to 2 eV, significant peak overlap is observed among Ti_3_C_2_ (red), Cu_3_(HHTP)_2_ (blue), and Si (green), indicating strong electronic coupling between these three components. This overlap suggests an efficient charge transfer pathway within the heterojunction, where Cu_3_(HHTP)_2_ serves as an intermediate charge transport layer, facilitating stepwise electron transfer between Si and Ti_3_C_2_. The observed hybridization between the states of Cu_3_(HHTP)_2_ and Ti_3_C_2_ in this energy range implies that Cu_3_(HHTP)_2_ plays a crucial role in modulating the interfacial electronic structure, reducing charge recombination, and improving carrier transport efficiency. Additionally, the broad distribution of states in this region, particularly from Ti_3_C_2_, indicates that Ti_3_C_2_T_x_ contributes to an extended charge transport channel, enhancing the conductivity of the heterostructure. Thus, the synergistic effect of Cu_3_(HHTP)_2_ and Ti_3_C_2_T_x_, confirmed by the strong DOS overlap in the −2 to 2 eV range, enables efficient charge transfer, reduces energy barriers for carrier migration, and significantly enhances the device's overall photoelectric performance. This analysis clearly delineates the distinct yet complementary roles of Cu_3_(HHTP)_2_ as a hole transport layer and Ti_3_C_2_T_x_ as an electron‐extracting Schottky electrode, leading to superior light response characteristics in the Ti_3_C_2_/Cu_3_(HHTP)_2_/*n*‐Si heterojunction.

**Figure 4:**
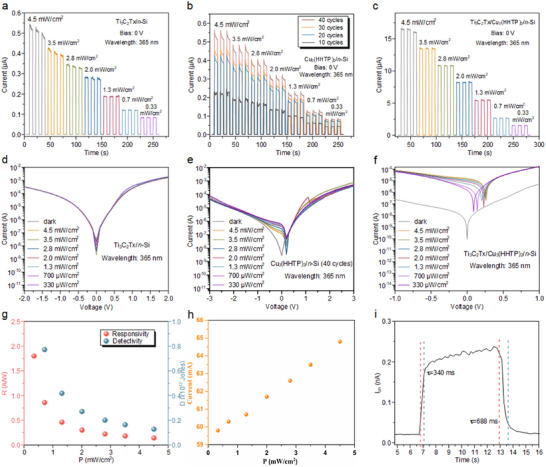
Self‐powered photodetection performance of the Ti_3_C_2_T_x_/Cu_3_(HHTP)_2_/*n*‐Si photodetector. The I–T characteristic curves of a) Ti_3_C_2_T_x_/*n*‐Si, b) Cu_3_(HHTP)_2_/*n*‐Si with different thicknesses, and c) Ti_3_C_2_T_x_/Cu_3_(HHTP)_2_/*n*‐Si photodetectors at different optical power densities at 365 nm wavelength; The I–V logarithmic curves of d) Ti_3_C_2_T_x_/*n*‐Si, e) Cu_3_(HHTP)_2_‐40C/*n*‐Si, and f) Ti_3_C_2_T_x_/Cu_3_(HHTP)_2_/*n*‐Si photodetectors; g) Linear dynamic range curve, h) responsivity and detection rate, and i) response time for Ti_3_C_2_T_x_/Cu_3_(HHTP)_2_/*n*‐Si photodetector.

To investigate the performance of self‐powered photodetectors, Ti_3_C_2_T_x_/*n*‐Si, Cu_3_(HHTP)_2_/*n*‐Si, and Ti_3_C_2_T_x_/Cu_3_(HHTP)_2_/*n*‐Si were fabricated and their photodetection performance was measured. The possible working mechanisms of these detectors were also explored. When Ti_3_C_2_T_x_ thin‐film contacts *n*‐Si, a Schottky energy barrier forms at the interface, causing electrons to transfer from Ti_3_C_2_T_x_ to *n*‐Si and creating a depletion region on the *n*‐Si side. This results in an upward band bending at the heterojunction interface, generating an internal electric field that is dependent on the work function difference between Ti_3_C_2_T_x_ and *n*‐Si. **Figure** **4**a shows the current time (I–T) response of the self‐powered photodetectors at a wavelength of 365 nm, under varying power densities (4.5 to 330 mW cm^−2^), at 0 V bias voltage, with a time 6s interval between light on and off. As the light intensity increases, the photocurrent increases accordingly, exhibiting good repeatability and stability. Figure 4d presents the I–V curves of the Ti_3_C_2_T_x_/*n*‐Si under dark conditions and 365 nm illumination (4.5 to 330 µW cm^−2^). Without illumination, the device showed a dark current of 0.0271 A at −2 V, while under 365 nm light, the photocurrent increased to 0.02942 A. The calculated light‐to‐dark current ratio was 1.08 at 365 nm and 4.5 mW cm^−2^, indicating substantial photocurrent generation. However, a high surface density of states on the Si interface pinned the Fermi level and increased charge recombination, resulting in a significant dark leakage current, which negatively impacted the photodetector's response and detection rates.^[^ ^53^ ^]^

Figure 4b,e, and Figure S11 (Supporting Information) present the I–T and I–V curves of the Cu_3_(HHTP)_2_/*n*‐Si photodetector with different thicknesses under 365 nm light and varying optical power densities. Compared with the Ti_3_C_2_T_x_/*n*‐Si, Cu_3_(HHTP)_2_/*n*‐Si photodetectors encountered similar issues. The higher resistivity of the 2D MOFs led to increased photocurrent and dark current. Cu_3_(HHTP)_2_/*n*‐Si with different thicknesses exhibited comparable optical performance. The elevated dark currents mainly stem from two factors: (I) In the Schottky barrier plus a reverse bias voltage, the applied voltage makes its depletion layer wider, the built‐in electric field becomes larger, the potential energy of the electron increases, the majority of the carriers in the P and N regions are very difficult to cross the barrier, and therefore the diffusion current tends to zero, but due to the increase in the junction electric field, which makes it easier to produce the drift movement of a minority of the carriers in the P and N regions, which generates a dark current. (II) Crystal defects in Cu_3_(HHTP)_2_ can play the role of the composite center, can capture holes and electrons, so that they are composite, the composite process is always accompanied by the directional movement of the carriers, there is bound to be a small dark current. The results demonstrate that, despite the device exhibiting a substantial photocurrent in practical operation, the high density of surface states on the Si substrate pins the Fermi level at the interface. This enhances interfacial charge recombination, leading to a significant increase in dark leakage current, which severely degrades the photodetector's responsivity and detectivity. In summary, both Ti_3_C_2_T_x_/*n*‐Si and Cu_3_(HHTP)_2_/*n*‐Si photodetectors suffer from significant dark current issues, which impede their performance.

To verify the stability of Ti_3_C_2_T_x_/Cu_3_(HHTP)_2_/*n*‐Si photodetectors, I‐T curves were collected during self‐powered operation. Figure 4c shows the I–T curves for the Ti_3_C_2_T_x_/Cu_3_(HHTP)_2_/*n*‐Si device at varying optical power densities (330 µW cm^−2^ to 4.5 mW cm^−2^) with 365 nm wavelength and 0 V bias. As the light intensity increased, the photoelectric current rose proportionally, while the noise level of the photocurrent remained consistent, demonstrating excellent repeatability and stability. As shown in Figure 4f, the I–V test results of the Ti_3_C_2_T_x_/Cu_3_(HHTP)_2_/*n*‐Si photodetector were tested in both dark conditions and 365 nm wavelength illumination (330 µW cm^−2^ to 4.5 mW cm^−2^). The I–V curves show that the photocurrent decreases with a reduction in negative bias voltage; the photocurrent increases with the increase of optical power under the same bias voltage and increases with higher optical power at the same bias voltage. In the absence of illumination, the device exhibited a dark current of 3.47 × 10^−6^ A at −1 V and 8.71 × 10^−11^ A at 0 V. Under 365 nm illumination with a power density of 4.5 mW cm^−2^, the maximum photocurrent was 6.48 × 10^−4^A at −1 V, and the minimum photocurrent at 0 V was 3.4 × 10^−6^ A. The maximum photocurrent‐to‐dark‐current ratio at 0 V was calculated to be 3.9 × 10^4^, indicating significant sensitivity to 365 nm wavelength incident light. The generation of electron‐hole pairs under light irradiation effectively contributed to the photocurrent. Further analysis of the I–V curve revealed a prominent photovoltaic effect, confirming that the Ti_3_C_2_T_x_/Cu_3_(HHTP)_2_/*n*‐Si photodetector can operate autonomously at 0 V bias without the need for an external power supply.

**Figure 5:**
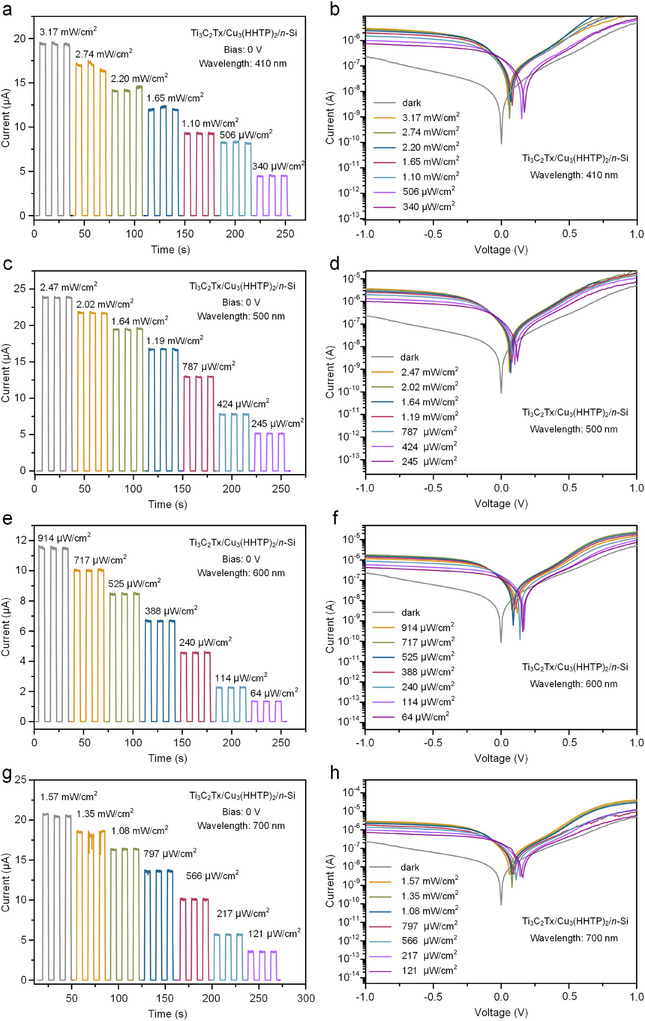
The I–T curves of Ti_3_C_2_T_x_/Cu_3_(HHTP)_2_/*n*‐Si photodetector at different optical power densities at a) 410 nm, c) 500 nm, e) 600 nm, and g) 700 nm wavelengths. Corresponding; The I–V curves for at b) 410 nm, d) 500 nm, f) 600 nm, and h) 700 nm wavelengths.

Responsivity and detectability are key parameters for evaluating the sensitivity and detection capability of photodetectors, respectively. As shown in Figure 4g, the photoresponsivity and detectability curves of the Ti_3_C_2_T_x_/Cu_3_(HHTP)_2_/*n*‐Si photodetector under zero bias voltage demonstrate that the detector's responsivity increases with higher optical power density. After calculation, the maximum responsivity (R) reached approximately 1.8 A W^−1^, while the detection (D*) was 1.63 × 10^12^ Jones at an optical power density of 330 µW cm^−2^. The linear dynamic range reflects the quantitative relationship between the incident light intensity and the generated photocurrent. As depicted in Figure 4h, the maximum linear dynamic range of the Ti_3_C_2_Tx/Cu_3_(HHTP)_2_/n‐Si device was 69.7 dB with various power intensity ranging from dark to 4.5 mW cm^−2^, highlighting the device's ease of operation within its linear range and its solid foundation for quantization. Figure 4i shows the switching cycle response time at a 365 nm wavelength with an optical power density of 4.5 mW cm^−2^. The rise and fall times were measured to be 340 ms and 688 ms, respectively. To further investigate the response of the Ti_3_C_2_T_x_/Cu_3_(HHTP)_2_/*n*‐Si photodetector to various wavelengths of incident light, its photo response was explored at wavelengths ranging from 410 to 700 nm. **Figure** **5** shows the I–T response and I–V characteristic of the photodetector under different wavelengths, and Table S2 (Supporting Information) summarizes the device's performance parameters under these conditions. The results indicate that the device exhibits high responsivity, detectivity, and switching ratio under UV and near‐infrared wavelengths. The Ti_3_C_2_T_x_/Cu_3_(HHTP)_2_/*n*‐Si photodetector not only operates in a self‐powered mode but also achieves low dark current due to the absence of external voltage. Furthermore, the device exhibits significant optical response rates, high photovoltaic conversion efficiencies and low noise equivalent power (NEP) across the wavelength range of 365 nm to 700 nm, with the best performance observed at 365 nm. The device shows optimal performance at specific wavelengths rather than a monotonic change, likely attributed to the nonlinear variation of the absorption coefficient with wavelength, with each sub‐band efficiently responding to its corresponding spectral range, resulting in overall multi‐peak performance due to differences in carrier collection efficiency.

## 3 Conclusion

This work reported an all‐solution processable fabrication approach for fabricating Ti_3_C_2_T_x_/Cu_3_(HHTP)_2_/*n*‐Si heterojunction as self‐powered photodetectors through the consecutive spray coating a Cu_3_(HHTP)_2_ and Ti_3_C_2_T_x_ thin films on *n*‐type silicon surface, as formed self‐supplied photodetector reveals high responsivity and detectivity. By utilizing the potential barrier difference between the Cu_3_(HHTP)_2_/*n*‐Si type I heterojunction and the Ti_3_C_2_T_x_/Cu_3_(HHTP)_2_ Schottky junction, photogenerated electrons are efficiently transported from Ti_3_C_2_T_x_ through the Cu_3_(HHTP)_2_ layer to *n*‐Si, significantly increasing the photocurrent while reducing the dark current. The Ti_3_C_2_T_x_/Cu_3_(HHTP)_2_/*n*‐Si photodetector demonstrates exceptional photoresponse performance, with a responsivity of 1.8 A W^−1^, a specific detectivity of 1.63 × 10^12^ Jones, and a switching ratio of 3.9 × 10^4^ at zero bias at a power density of 330 µW cm^−2^ of incident light. Additionally, it delivers excellent photoresponse performance across wavelengths from 365 to 700 nm. This work underscores the feasibility of integrating 2D MOFs and MXene thin films into broadband high‐performance photodetectors via an all‐solution processable method. By addressing interfacial defects through band design and structural optimization, this work paves the way for new applications and innovations in optoelectronics.

## 4 Experimental Section

### Materials Preparation

Copper acetate, hydrochloric acid, and lithium fluoride were purchased from Energy Chemical (Shanghai) Co., Ltd. 2,3,6,7,10,11‐Hexahydroxybenzobenzene (98% purity) was purchased from Meryer (Shanghai) Chemical Technology Co., Ltd. Acetone, isopropanol, methanol, and ethanol were bought from Sinopharm Chemical Reagent Co., Ltd. Ti_3_AlC_2_ (98%) was sourced from Shandong Shenyan New Materials Technology Co., Ltd. All reagents were used without further purification treatment, and deionized water (18.2 MΩ•cm) was used for all aqueous solutions throughout the experimental process.

### Preparation of Cu_3_(HHTP)_2_/*n*‐Si

Two‐dimensional conductive Cu_3_(HHTP)_2_ thin films were grown layer‐by‐layer onto an *n*‐type silicon (*n*‐Si) substrate. The monocrystalline silicon wafer (resistivity: 1–2.5 Ω m) was alternatively sprayed with methanol solutions containing 0.04 mM copper acetate (Cu(OAc)_2_·H_2_O) and 0.02 mM 2,3,6,7,10,11‐Hexahydroxytriphenylenehydrate (HHTP). Each solution was alternately sprayed for 2 min–, with a rinse using ethanol to remove unreacted reactants between sprays, followed by drying. The thickness of thin films was controlled by varying the number of deposition cycles. Cu_3_(HHTP)_2_ thin films with different thicknesses were labeled as Cu_3_(HHTP)_2_‐*x*C, where *x* (*x* = 10, 20, 30, 40) denotes the number of deposition cycles.

### Preparation of Ti_3_C_2_T_x_ Mxenes Nanosheets

Hydrochloric acid (40 mL of 9 M) was added into 100 mL of polytetrafluoroethylene liner, followed by 2 g of lithium fluoride and 2 g of Ti_3_AlC_2_, which were gradually introduced into the solution. The mixture was stirred for 1 h in an ice bath. The black suspension was obtained by further stirring at 35 °C for 48 h. The suspension was then centrifuged and repeatedly washed with deionized water until the pH of the supernatant reached 6, yielding the black powder. The black powder, along with 160 mL ethanol, was put into a 250 mL PTFE liner, injected with nitrogen gas, and sonicated in an ice bath for 2 h. After centrifugation at 10 000 rpm for 30 min, the upper black liquid was collected as Ti_3_C_2_T_x_ nanosheet suspension with a concentration of approximately 5 mg/mL.

### Preparation of Ti_3_C_2_/Cu_3_(HHTP)_2_/*n*‐Si

The Cu_3_(HHTP)_2_ was grown layer by layer on the *n*‐Si substrate. The prepared 2D Ti_3_C_2_T_x_ nanosheets were then sprayed onto the Cu_3_(HHTP)_2_ layer to form the Ti_3_C_2_T_x_/Cu_3_(HHTP)_2_/*n*‐Si heterojunctions. Specifically, the Ti_3_C_2_T_x_ nanosheet suspension was loaded into a spray gun, and the Cu_3_(HHTP)_2_/*n*‐Si thin film was fixed on an upright backplate. Using the centerline of the Cu_3_(HHTP)_2_/*n*‐Si substrate as the axis for masking, Ti_3_C_2_T_x_ Mxenes were sprayed onto the exposed surface of the MOF thin film. The sprayed silicon wafers samples were placed in a vacuum drying oven at 60 °C and dried prior to measurement.

### Characterization and Measurements

The morphological characteristics and element distribution of Cu_3_(HHTP)_2_ thin films and 2D Ti_3_C_2_T_x_ Mxenes nanosheets were studied using optical imaging (OM), scanning electron microscopy (SEM), and energy dispersive X‐ray spectroscopy (EDS). The chemical and crystal structures of Cu_3_(HHTP)_2_ thin films and 2D Ti_3_C_2_ Mxenes nanosheets were characterized using infrared spectroscopy, X‐ray photoelectron spectroscopy (XPS), X‐ray diffraction (XRD), and transmission electron microscopy (TEM). The band structure of Cu_3_(HHTP)_2_ thin films was analyzed using UV–vis diffuse reflection and Mott–Schottky spectroscopy.

## Conflict of Interest

The authors declare no conflict of interest.

## Supporting information



Supporting Information

## Data Availability

The data that support the findings of this study are available from the corresponding author upon reasonable request.
