# Supplementary material for: Solution‐Processable Van Der Waals Heterojunctions on Silicon for Self‐Powered Photodetectors with High Responsivity and Detectivity
Source: Adv Sci (Weinh). 2025 Mar 28;12(23):2500027. doi: 10.1002/advs.202500027 (PMC12199377; doi:10.1002/advs.202500027)
Supplement: Supplementary file 1 — Supporting Information [file ADVS-12-2500027-s001.docx]

Supporting Information

Solution-processable van der Waals Heterojunctions on Silicon for Self-powered Photodetectors with High Responsivity and Detectivity

**Table S1.** Comparison of performance parameters for various electrodes and attempts at using MXene or MOF materials in self-powered photodetectors.

| Devices | Wavelength (nm) | Responsivity (A/W) | Detection (Jones) | I_on_/I_off_ | ref |
| --- | --- | --- | --- | --- | --- |
| Ti_3_C_2_T_x_/Cu_3_(HHTP)_2_/n-Si | 365 | 1.8 | 1.63×10^12^ | 3.9×10^4^ | This work |
| Cu_3_(HHTP)_2_/ZnO | 365 | 1.1×10^-3^ | 1.31×10^8^ | 8.0×10^3^ | 1 |
| ZrBDC/SnO_2_ | 254 | 1.7×10^-3^ | 7.85×10^10^ | 3.8×10^2^ | 2 |
| ZnO/Ni-CAT-3 | 450 | 1.4×10^-4^ | / | 1.0×10^2^ | 3 |
| Ag/Ni-HITP/Si | 450 | 0.3 | 3.20×10^11^ | 5.3×10^2^ | 4 |
| Eu−MOF | 254 | 2.8×10^-4^ | 1.02×10^10^ | 1.1×10^2^ | 5 |
| ZnTCPP MOF | 520 | 0.27 | 4.61×10^12^ | 1.0×10^4^ | 6 |
| TiO_2_-NiFeMOF@CNTF | 375 | 0.089 | 3.81×10^13^ | / | 7 |
| Ti_3_C_2_-MXene/ZnO | 368 | 0.14 | 2.0×10^10^ | 8.1×10^2^ | 8 |
| Fe_3_(THT)_2_(NH_4_)_3_ MOF | 785 | 4.0×10^-3^ | 7.0×10^8^ | / | 9 |
| PEDOT:PSS/Ga_2_O_3_ | 284 | 0.037 | 9.2×10^12^ | 8.0×10^2^ | 10 |
| Ti_3_C_2_T_x_/GaN | 355 | 0.28 | 7.06×10^13^ | / | 11 |
| C_14_H_31_O_3_P-Ti_3_C_2_/Au | 1064 | 0.28 | 4.3×10^7^ | 10.4 | 12 |
| Ti_3_C_2_T_X_/In_0.15_Ga_0.85_N | 470 | 6.1 | 8.9×10^11^ | / | 13 |
| ZIF-8@H:ZnO NRs/p-Si | 365 | 0.21 | 6.35×10^13^ | / | 14 |
| ZnO-BiOCl/Ti_3_C_2_T_x_ | 350 | 9.4×10^-5^ | 5.86×10^10^ | 8.0×10^3^ | 15 |
| Ti_3_C_2_T_x_/NiO/TiO_2_/FTO | 365 | 0.020 | 4.1×10^10^ | / | 16 |
| Cu-HBC | 365 | 5.4×10^-5^ | / | 4.2×10^3^ | 17 |


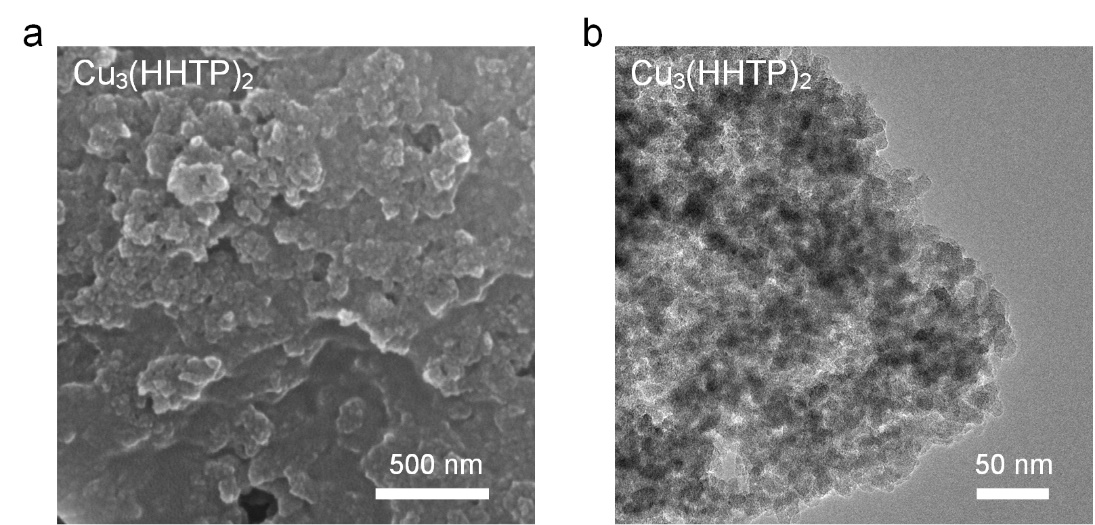


**Figure S1.** (a) SEM image and (b) TEM image of Cu_3_(HHTP)_2_-40C thin films.


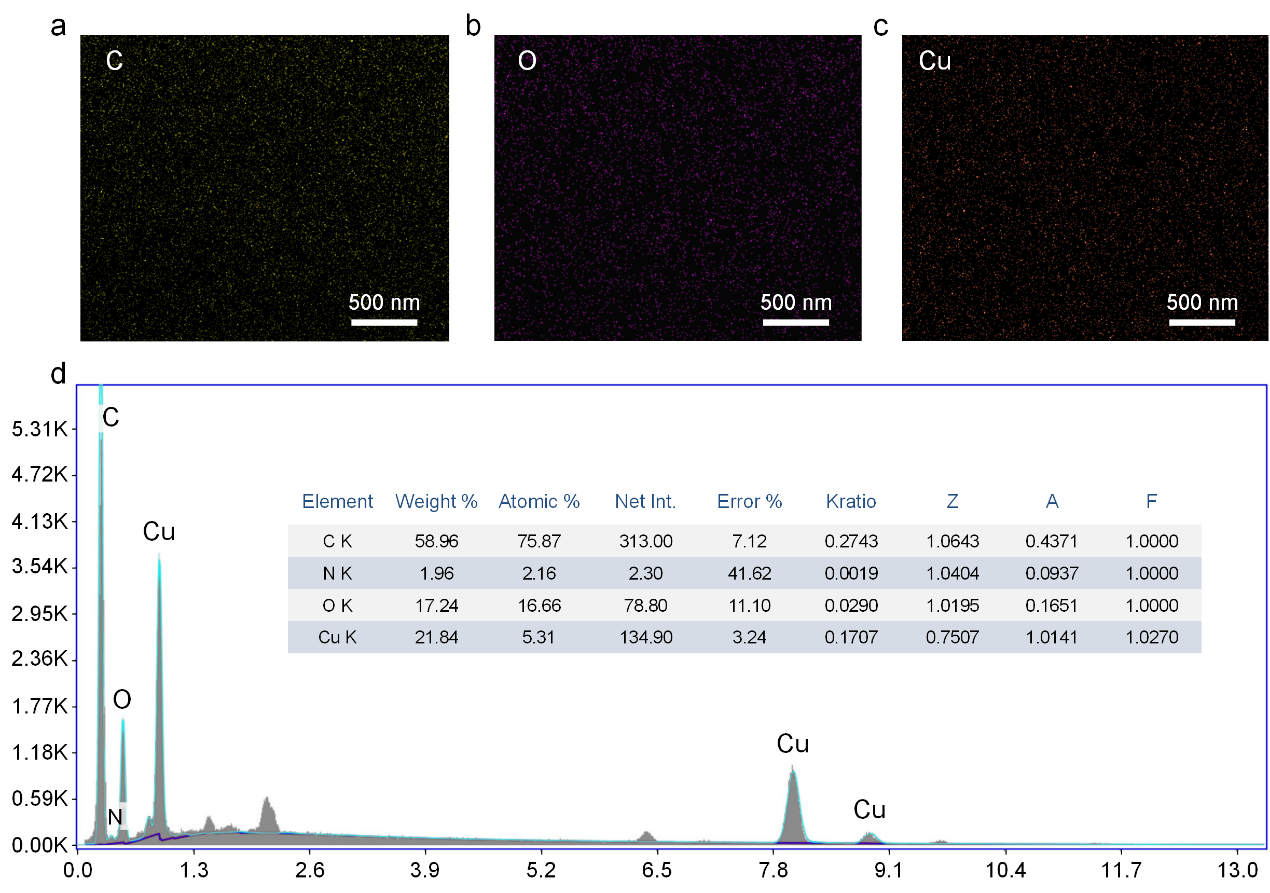


**Figure S2.** EDS mapping of elemental distribution (b) carbon, (c) oxygen, (d) copper, and (e) overall elemental distribution of the Cu_3_(HHTP)_2_-40C thin film.


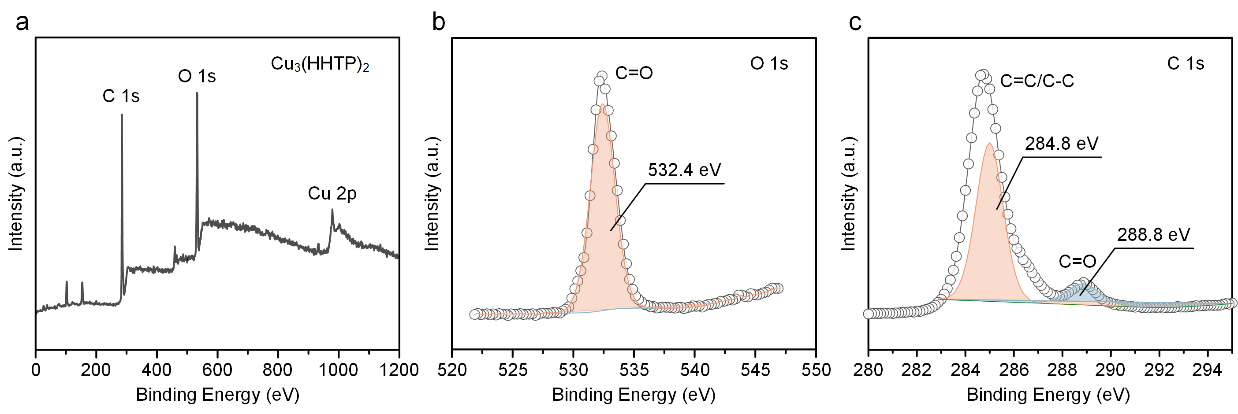


**Figure S3.** (a) XPS full spectrum, high-resolution XPS of (b) O 1s and (c) C 1s spectra of the Cu_3_(HHTP)_2_ thin film.


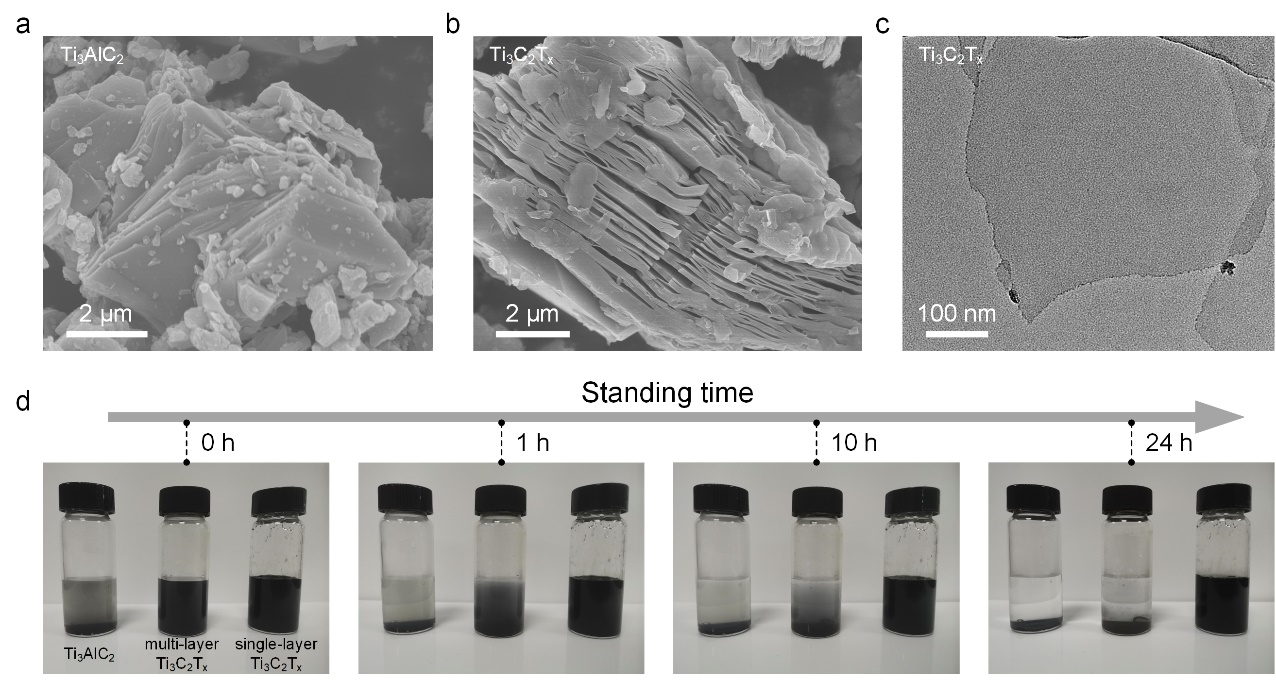


**Figure S4.** SEM images of (a) Ti_3_AlC_2_ precursor, (b) exfoliated multi-layer Ti_3_C_2_T_x_, and (c) single-layer Ti_3_C_2_T_x_. (d) The lower panels show optical images of Ti_3_AlC_2_, multi-layer Ti_3_C_2_T_x_, and single-layer Ti_3_C_2_T_x_ dispersed in water and left to stand in air for various durations.


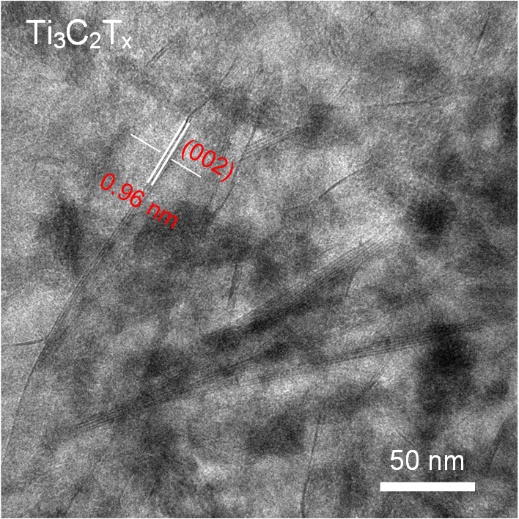


**Figure S5.** HR-TEM image of Ti_3_C_2_T_x_.


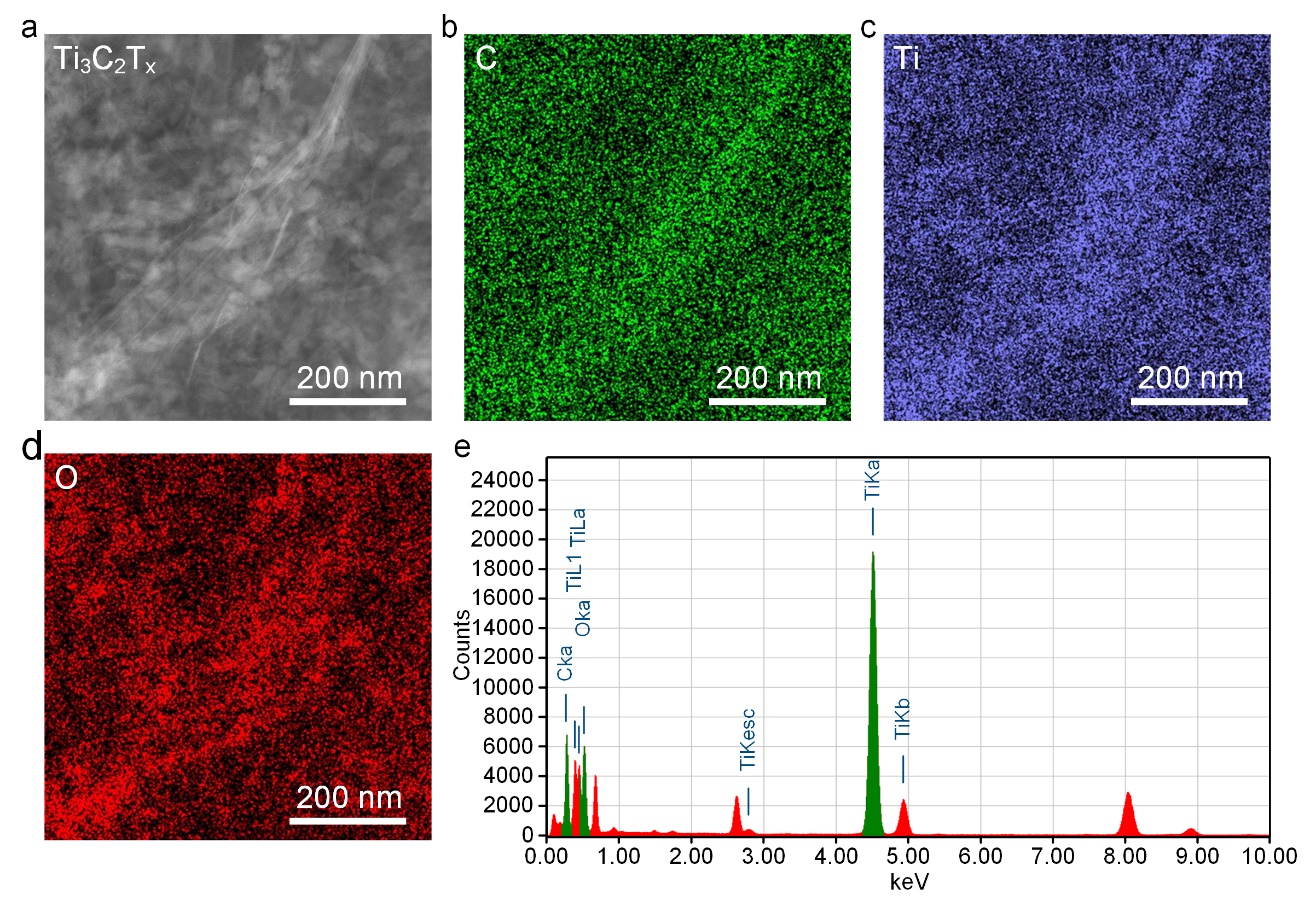


**Figure S6.** (a) TEM image of Ti_3_C_2_T_x_, and EDS mapping showing elemental distribution of (b) carbon, (c) titanium, (d) oxygen, and (e) elemental quantification analysis of Ti_3_C_2_T_x_.


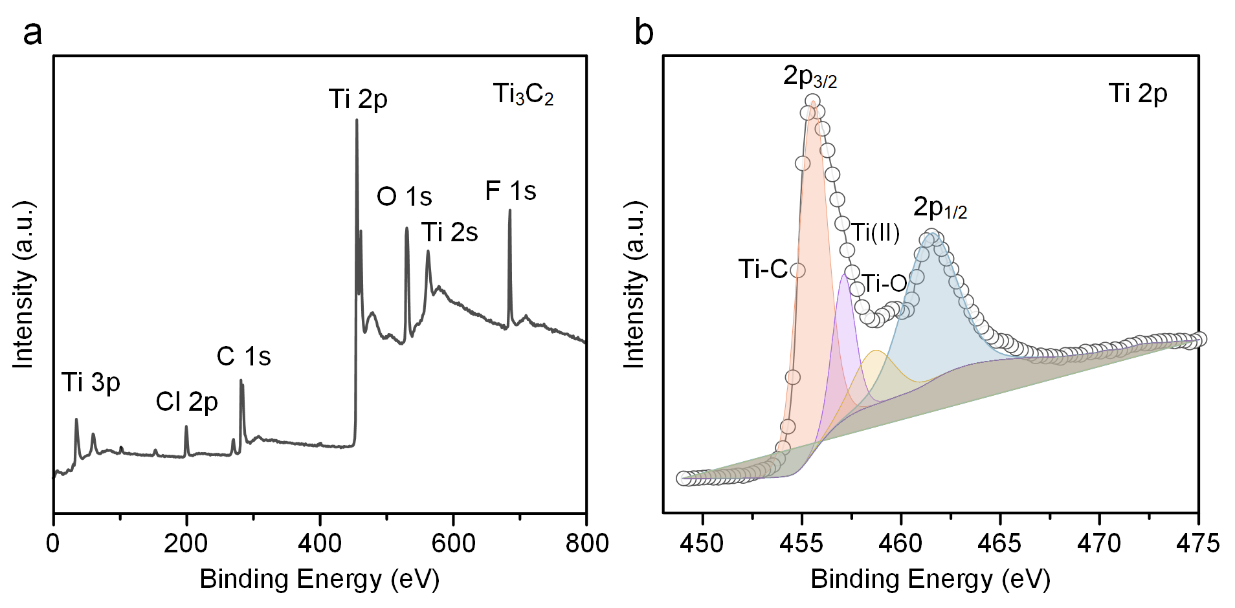


**Figure S7.** (a) XPS full spectrum and (b) high-resolution XPS Ti 2p spectrum of Ti_3_C_2_T_x_.


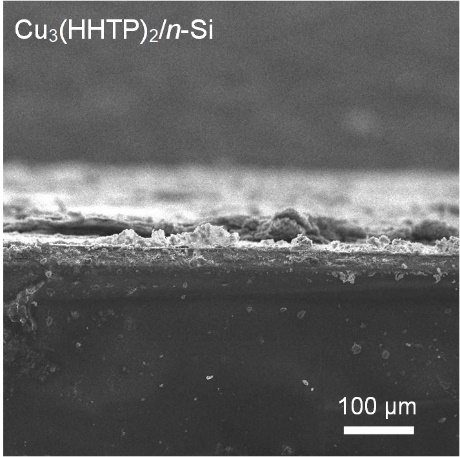


**Figure S8.** SEM images of Cu_3_(HHTP)_2_/*n*-Si cross-section.


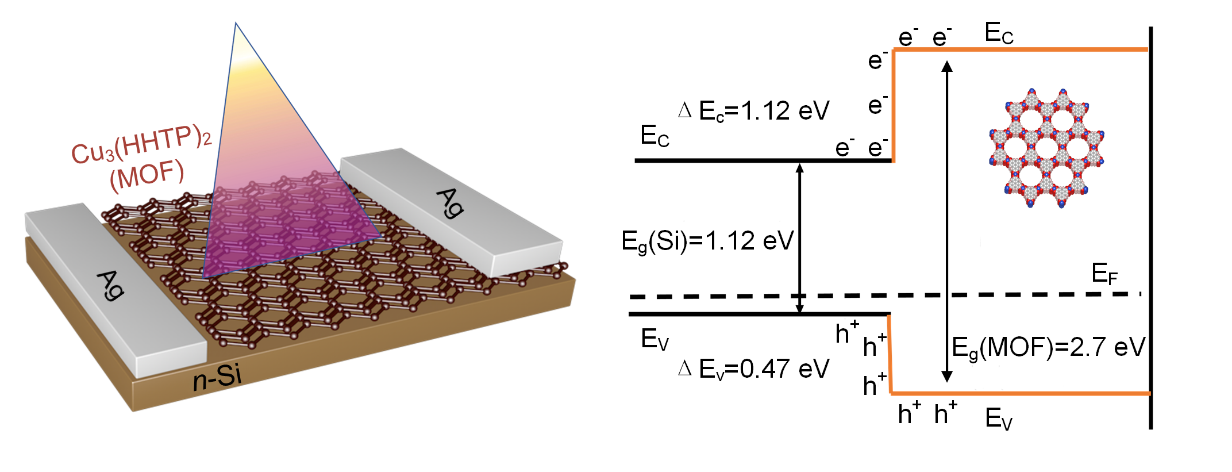


**Figure S9.** Schematic diagram and band structure diagram of Cu_3_(HHTP)_2_/*n*-Si photodetector.


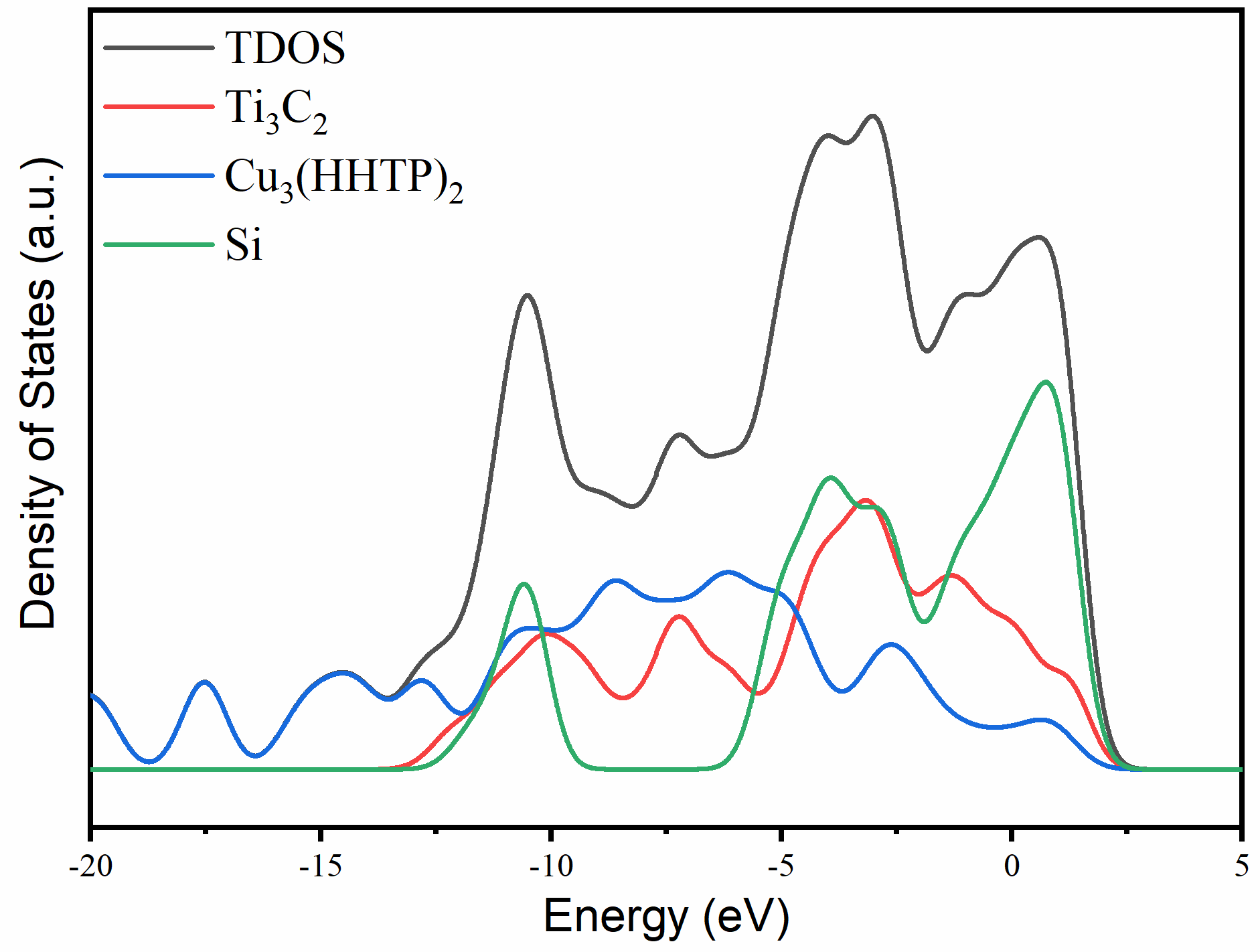


**Figure S10.** The density of states (DOS) for the Ti_3_C_2_/Cu_3_(HHTP)_2_/*n*-Si heterostructure.


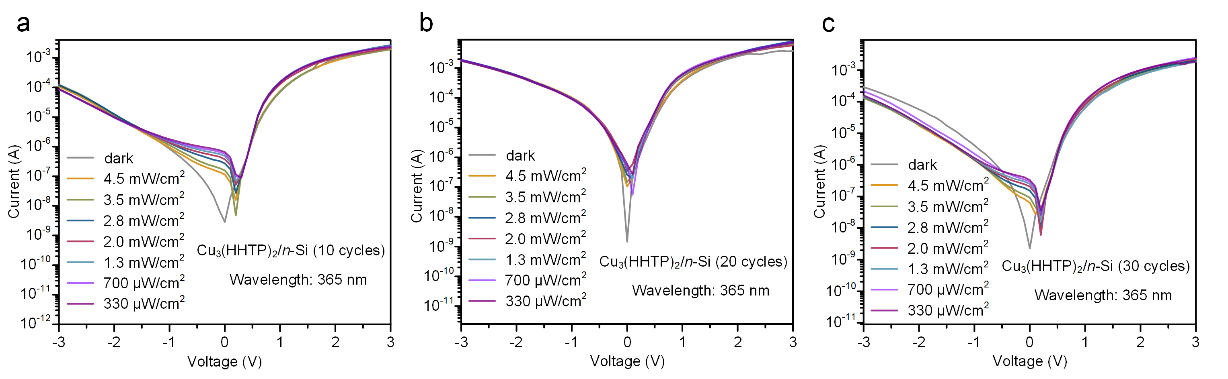


**Figure S11.** Logarithmic I-V curves of (a) Cu_3_(HHTP)_2_-10C/*n*-Si, (b) Cu_3_(HHTP)_2_-20C/*n*-Si, (c) Cu_3_(HHTP)_2_-30C/*n*-Si photodetectors under 365 nm wavelength irradiation at different optical power densities.

**Table S2.** Performance parameters of the Ti_3_C_2_T_x_/Cu_3_(HHTP)_2_/*n*-Si self-powered photodetector under irradiation at different light wavelengths.

| Wavelength (nm) | Responsivity  (A/W) | Detection  (Jones) | I_on_/I_off_ | NER |
| --- | --- | --- | --- | --- |
| 365 | 1.8 | 1.63×10^12^ | 3.9×10^4^ | 6.13×10^-13^ |
| 410 | 0.27 | 3.57×10^11^ | 1.0×10^3^ | 2.8×10^-12^ |
| 500 | 0.15 | 1.98×10^11^ | 9.4×10^2^ | 5.05×10^-12^ |
| 600 | 0.91 | 1.20×10^12^ | 1.68×10^3^ | 8.33×10^-13^ |
| 700 | 0.45 | 5.96×10^11^ | 9.9×10^2^ | 1.68×10^-12^ |

The calculation formulas of responsivity (R) and detection (D*) of the device are as follows:


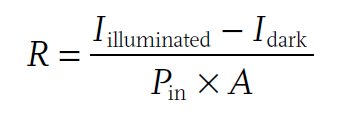


Where: I_illuminated​_ is the current (or voltage) measured when the device is illuminated. I_dark_​ is the current (or voltage) measured in the dark, with no light falling on the device. P_in_​ is the input optical power of the light source. A is the area of the device that is exposed to light.


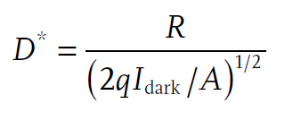


Where: *R* is the responsivity, as calculated above. *q* is the elementary charge (approximately 1.6×10^-19^ coulombs). *I*_dark_​ is the dark current, which is the current when no light is incident on the device. *A* is the active area of the device.

The detailed theoretical computational methodology is shown below:

The CP2K software was employed for the first-principles calculations. Initially, structural optimization was performed separately for the Ti_3_C_2_ layer, Cu-MOF layer, and Si layer, using the semi-empirical GFN1-xTB method combined with the Orbital Transformation (OT) diagonalization technique, with SMEAR enabled. After obtaining the optimized structures, model stacking was conducted using the Materials Studio (MS) software, forming three heterostructure models. Subsequently, geometry optimization of the heterostructures was performed using the PBE0 functional with DFT-D3(BJ) dispersion corrections and the MOLOPT DZVP-GTH basis set, under a 2×2×1 K-point grid. After optimization, single-point energy calculations were conducted on the final structures using a 3×3×1 K-point grid, from which wavefunction and orbital files were extracted for electronic density difference and DOS analysis.

**References**

[1] C. Kang, M. Ahsan Iqbal, S. Zhang, X. Weng, Y. Sun, L. Qi, W. Tang, S. Ruan, Y. J. Zeng, *Chem. Eur. J.* **2022**, *28*, e202201705.

[2] M. H. Tran, J. Hur, *Adv. Opt. Mater.* **2021**, *10*, 2101404.

[3] Y. Wang, L. Liu, Y. Shi, S. Li, F. Sun, Q. Lu, Y. Shen, S. Feng, S. Qin, *ACS Appl. Mater. Interfaces* **2023**, *15*, 18236.

[4] L.-A. Cao, M.-S. Yao, H.-J. Jiang, S. Kitagawa, X.-L. Ye, W.-H. Li, G. Xu, *J. Mater. Chem. A* **2020**, *8*, 9085.

[5] T. M. H. Nguyen, C. W. Bark, *ACS Appl. Mater. Interfaces* **2022**, *14*, 45573.

[6] M. Joshi, S. Sridhar, A. K. Sahu, V. K. Singhal, B. Kumar, *ACS Appl. Nano Mater.* **2023**, *6*, 22784.

[7] J. Zhou, L. Chen, J. Wu, Z. Lu, F. Liu, X. Chen, P. Xue, C. Li, L. Wei, G. Wu, Q. Li, Q. Zhang, *Nano Lett.* **2023**, *23*, 11297.

[8] T. Zheng, W. Wang, Q. Du, X. Wan, Y. Jiang, P. Yu, *ACS Appl. Nano Mater.* **2024**, *7*, 3050.

[9] H. Arora, R. Dong, T. Venanzi, J. Zscharschuch, H. Schneider, M. Helm, X. Feng, E. Cánovas, A. Erbe, *Adv. Mater.* **2020**, *32*, 1907063.

[10] S. Li, Z. Yan, Z. Liu, J. Chen, Y. Zhi, D. Guo, P. Li, Z. Wu, W. Tang, *J. Mater. Chem. C* **2020**, *8*, 1292.

[11] W. Song, J. Chen, Z. Li, X. Fang, *Adv. Mater.* **2021**, *33*, 2101059.

[12] L.-J. Du, J.-W. Chen, R.-M. Wang, *Acta Phys. Sin.* **2023**, *72*, 128502.

[13] D. Kong, T. Lin, J. Chai, Z. Zhu, P. Liu, Z. Lin, T. Lin, L. Li, L. Chen, G. Li, W. Wang, *Appl. Phys. Lett.* **2023**, *122*, 142104.

[14] T. Guo, C. Ling, X. Li, X. Qiao, X. Li, Y. Yin, Y. Xiong, L. Zhu, K. Yan, Q. Xue, *J. Mater. Chem. C* **2019**, *7*, 5172.

[15] W. Ouyang, J. Chen, J. H. He, X. Fang, *Adv. Electron. Mater.* **2020**, *6*, 2000168.

[16] T. T. Nguyen, G. Murali, M. Patel, S. Park, I. In, J. Kim, *ACS Appl. Energy Mater.* **2022**, *5*, 7134.

[17] Z. Xu, Y. Liu, A. Chandresh, P. B. Pati, V. Monnier, L. Heinke, F. Odobel, S. Diring, R. Haldar, C. Wöll, *Adv. Funct. Mater.* **2023**, *34*, 2308847.
